# Antiplatelet, Anticoagulant, and Fibrinolytic Activity of Orchids: A Review

**DOI:** 10.3390/molecules29235706

**Published:** 2024-12-03

**Authors:** Berenice Fernández-Rojas, Abimael López-Pérez, Luicita Lagunez-Rivera, Rodolfo Solano, Anel Karina Bernal-Martínez, Abraham Majluf-Cruz, Jesús Hernández-Juárez

**Affiliations:** 1 Laboratorio de Nutracéuticos y Productos Naturales, Centro de Estudios en Ciencias de la Salud y la Enfermedad, Facultad de Odontología, Universidad Autónoma Benito Juárez de Oaxaca, Oaxaca 68120, Mexico; berenicefdezr@gmail.com; 2 Centro Interdisciplinario de Investigación para el Desarrollo Integral Regional Unidad Oaxaca, Instituto Politécnico Nacional, Oaxaca 71233, Mexico; ablopezp@ipn.mx (A.L.-P.); llagunez@ipn.mx (L.L.-R.); asolanog@ipn.mx (R.S.); abernalm@ipn.mx (A.K.B.-M.); 3 Unidad de Investigación Médica en Trombosis, Hemostasia y Aterogénesis, Hospital General Regional No. 1 Dr. Carlos Mac Gregor Sánchez Navarro, Instituto Mexicano del Seguro Social, Mexico City 031013, Mexico; amajlufc@gmail.com; 4 CONAHCYT-Centro Interdisciplinario de Investigación para el Desarrollo Integral Regional Unidad Oaxaca, Instituto Politécnico Nacional, Santa Cruz Xoxocotlán 71233, Oaxaca, Mexico

**Keywords:** natural products, thrombosis, antithrombotic treatment

## Abstract

Thrombosis is the occlusion of a blood vessel and is responsible for the highest number of deaths worldwide. Its treatment comprises the use of anticoagulants, antiplatelets, and thrombolytics. Although many antithrombotic drugs are currently available, none is completely effective and safe. Plants are a valuable source of compounds with antithrombotic properties. Some orchid species have been used in traditional medicine for their antithrombotic properties. This review informs about the contribution of orchids in this field and the studies that have validated their properties.

## 1. Introduction

Blood coagulation is a complex physiological event that converges the principles of the cell-based and cascade coagulation models with the innate immune activation and inflammation in a unified host response [1]. In other words, the coagulation system requires hemostasis to work inseparably from innate immune and inflammatory processes in the physiological response to injury. Although the current knowledge of how a clot forms involves other processes, it is imperative to highlight that the main element for blood coagulation remains hemostasis, which, along with its natural regulatory mechanisms and fibrinolysis, allows clot formation perfectly located at injured vessels to stop blood loss [2]. Briefly, hemostasis is the system that drives clot formation in damaged blood vessels. It mainly involves the action of platelets and coagulation factors. Platelets in normal circulation are in a no-adherent resting state and become activated at sites of vascular injury after exposure to immobilized adhesive proteins such as collagen and von Willebrand factor (VWF) or soluble platelet agonists such as thrombin, epinephrine, ADP, thromboxane A_2_, and serotonin. Platelet activation comprises changes in their morphology, exposure of membrane phospholipids, release of granular content, and activation of the platelet receptor α*_IIb_*β_3_ that are conducive to platelet aggregation and, subsequently, clot retraction [3]. In a parallel event developing on the membrane of activated platelets, plasma hemostasis factors activate each other to form fibrin. It develops in three phases. The initiation phase occurs on the tissular factor-bearing cells after the exposition of sub-endothelium to the flowing blood. This phase aims to activate factors IX and X to generate small amounts of thrombin that diffuse away from cells of the sub-endothelium to the platelets. In the amplification phase, a small amount of thrombin activates platelets, releases VWF, and generates activated forms of factors V, VIII, and XI. Finally, in the propagation phase, the enzymes generated in earlier phases assemble on the procoagulant membrane surface of the activated platelet to form the intrinsic tenase and prothrombinase complexes, resulting in a burst of thrombin generation directly on the membranes of activated platelets, which converts the fibrinogen into fibrin. Once the fibrin/platelet clot has formed at the injury site, coagulation must be limited by the natural regulation systems to prevent widespread fibrin formation [4,5].

On the other hand, when the clot is no longer required, it must be removed. Fibrinolysis is the proteolytic degradation of the fibrin network that releases cellular components into the bloodstream. It is mainly regulated by two proteins, the tissue-type plasminogen activator (t-PA) and the urokinase-type plasminogen activator (u-PA), which convert the plasminogen into plasmin to initiate fibrin lysis; however, other factors can also contribute to plasmin generation. Fibrinolysis has inhibitory mechanisms that target specific fibrinolytic enzymes or impede the binding of fibrinolytic enzymes to fibrin [6].

After this simplified overview of hemostasis and fibrinolysis, it is also imperative to know that blood coagulation is just a transient event, because maintaining the liquid state of blood is necessary for life. On physiological conditions, the endothelium allows liquid blood within arterial and venous blood vessels and in the heart chambers, for which it performs three essential functions: antiplatelet, anticoagulant, and fibrinolytic. If the endothelium functions appropriately, then these functions are carried out adequately, preserving the liquid state of the blood and allowing life [2,7]. On the contrary, when the endothelium malfunctions, there is a tendency for the blood to abnormally turn into a solid, which is called a thrombus [7]. From an overall point of view, thrombosis is caused by pathological states, hereditary or acquired, primary or secondary, provoked or unprovoked, that affect the natural balance of hemostasis. These states include endothelial dysfunction, alterations in circulating anticoagulants, procoagulants, fibrinolytic factors [8,9,10], and blood stasis.

Thrombosis is defined as the local obstruction of blood flow in arteries or veins by a thrombus, allowing tissue ischemia that, if prolonged, causes irreversible cellular injury, affecting any organ [11]. Thrombosis is extremely lethal, mainly in arteries, which is responsible for one in four deaths worldwide, representing the leading cause of death in humans [12,13]. The scenario is equally discouraging for venous thromboembolism (VTE). As many as 900,000 people in the United States of America may be affected by VTE each year, with up to 100,000 dying as a result. For 25% of patients, the presenting manifestation of pulmonary embolism is sudden death [14].

The impact of thrombosis worldwide leads to millions of people being under some antithrombotic treatment (anticoagulants, antiplatelets, and/or fibrinolytics). Although a battery of antithrombotic drugs is available for patients, none of these is entirely effective and safe, and some others are hard to manage [15,16]. For these reasons, identifying and developing new agents with antithrombotic potential remains very important today. Plants offer a range of possibilities to understand the anticoagulant, antiplatelet, and fibrinolytic mechanisms of many bioactive compounds, which can be chemically modified to improve their antithrombotic properties [17].

Orchids are the most diverse group of flowering plants in the world, with approximately 28,000 known species, and they are found in almost every place on the planet [18]. These plants are known for their aesthetic qualities and are often used as decorative elements. For a long time, orchids have been used for medicinal purposes. Medicinal orchids have many therapeutic applications; they serve as analgesics, antidotes, anthelmintics, aphrodisiacs, emetics, and laxatives; they treat diabetes and other metabolic disorders, among other uses [19]. In Mexico, a recent publication informed about several orchids with apparent medical importance used to treat different human illnesses and diseases, including those with effects on circulation, hemorrhage, inflammation, wound healing, and heart conditions [20], effects that may be related to blood coagulation and its alterations. Despite the medicinal knowledge of orchids, it is important to highlight that only some have scientifically proven medicinal properties. In the field of thrombosis, the use of orchids has been documented, mainly their antiplatelet activity, but their anticoagulant and fibrinolytic activities have been little explored. Therefore, this research aimed to gain insight into the potential of natural orchid compounds with antiplatelet, anticoagulant, or fibrinolytic activity that may contribute to developing safer and more effective antithrombotic drugs than those currently available. Additionally, the review discusses some gaps that could be addressed in further studies and the possible direction that research in this field should follow, considering new trends in antithrombotic treatment.

## 2. Orchids with Antithrombotic Properties

The orchids that have been associated with antithrombotic activity in the literature are the following: *Dendrobium loddigesii* Rolfe., *Dendrobium densiflorum* Lindl., *Dendrobium longicornu* Lindl., *Dendrobium trigonopus* Rchb.f., *Dendrobium venustum* Teijsm. & Binn., *Cleisomeria lanatum* (Lindl.) Lindl. ex. G.Don, *Ephemerantha lonchophylla* (Hook. f.) P.F. Hunt & Summerh., *Calanthe arisanensis* Hayata, *Gastrodia elata* Blume, *Cephalanceropsis gracilis* (Lindl.) S.Y. Hu, and *Laelia furfuracea* Lindl. The findings for each of these species are described here. Due to the aim of this article, the information about the extraction, fractionation, identification, and purification of bioactive compounds must be consulted in the original papers. On the contrary, we dedicate a few lines to describe the laboratory tests used in orchid studies to evaluate their antithrombotic properties. Briefly, for the study of antiplatelet activity, the most used test described in the articles below was light transmission platelet aggregation, the gold standard for assessing platelet function [21,22]. This test measures platelet aggregation (PA) in real time using platelet-rich plasma (PRP) and specialized equipment. To induce PA, one of several platelet agonists (thrombin, ADP, collagen, arachidonic acid, among others) is added to PRP. Subsequently, after adding the agonist, the light transmission is measured in real time, and a graph is constructed. Finally, the percentage of PA is calculated [21,22]. The bleeding time (BT) in mice is an in vivo assay used for the overall analysis of primary hemostasis. This assay involves a longitudinal incision or transverse amputation of the tip of the tail. It is usually conducted at room temperature, either by blotting with filter paper every few seconds until bleeding ceases or dipping the tail into tubes in a preset time-frame to determine the duration of bleeding. It is common to immerse the tail in isotonic solution at 37 °C [23]. The plasma recalcification time (RT) is a screening test to evaluate the total coagulation mechanism. It assesses all plasma coagulation factors and platelet function. The principle is simple; the addition of warm (37 °C) calcium chloride (0.025 M) to a PRP allows for clot formation. Therefore, the time of clot formation following the addition of calcium chloride comprises the RT [24]. The whole-blood clotting time (CT) is a test performed directly in a glass tube to evaluate clot formation. The time to form a clot at 37 °C is recorded. The test assesses the activity of intrinsic and common coagulation factors. The activated CT uses an activator of the contact phase. The immediate activation of freshly drawn blood by an inert contact activator greatly shortens the coagulation time CT and produces a rather sensitive test for coagulation defects other than factor VII and platelet deficiencies [25]. In addition to CT, the prothrombin (PT), activated partial thromboplastin (APTT), and thrombin (TT) times were used to evaluate the anticoagulant effects of some orchids. Briefly, PT, APTT, and TT are evaluated in platelet-poor plasma (PPP). PT evaluated the activity of factor VII (extrinsic pathway) and factors from the common pathway (I, II, V, and X) in the presence of calcium and tissue factor. On the other hand, APTT evaluated the intrinsic activity (VIII, IX, XI, and XII) and common factors when an activator of the contact phase is added to the plasma. Finally, TT exclusively evaluates the conversion of fibrinogen into fibrin by adding exogenous thrombin [26]. Using these assays allows us to estimate a likely anticoagulant mechanism for the bioactive compounds of orchids. To explore the thrombolytic (fibrinolytic) activity of orchids, a commercial synthetic substrate of plasmin, the fluorophore AFC (7-amido-4-trifluoromethylcoumarin), was employed [27]. This test evaluated how the studied compounds stimulated the enzymatic activity of plasmin. Another way to evaluate the thrombolytic activity was to weigh the clot before and after adding the extract or the study compound. The difference obtained in the weight taken before and after the experimental condition was expressed as the lysis percentage.

### 2.1. Dendrobium loddigesii

Chen et al., in 1994 [28], showed in washed rabbit platelets that fractions of the methanolic extract of *D. loddigesii* stem inhibit platelet aggregation (PA). The light transmission platelet aggregometry method was used to measure PA. Platelets were activated with four agonists: thrombin (0.1 U/mL), arachidonic acid (AA: 100 µM), collagen (10 µg/mL), and platelet-activating factor (PAF: 2 ng/mL). Six fractions were obtained, but only fractions 3, 4, and 5 showed an inhibitory effect. Fraction 3 suppressed the collagen-induced PA (0.0 ± 0.0%) at 200 µg/mL concentration, while fraction 4 reached this effect at 50 µg/mL. In addition, fractions 4 (100 and 200 µg/mL) and 5 (200 µg/mL) inhibited the collagen-induced PA. At 200 µg/mL, fractions 4 and 5 decreased PA vs. control (0.5% DMSO), from 89.3 ± 0.9% to 7.6 ± 3.6% and from 89.3 ± 0.9% to 4.8 ± 3.9%, respectively. Subsequently, chromatographic analysis of fraction 4 was carried out because this one had the best inhibitory effect. Two compounds were identified: moscatilin (compound **1**) and moscatin (compound **2**). Acetylation of moscatilin led to the third compound. The effect of compounds **1**, **2**, and **3** on thrombin-, AA-, collagen-, and PAF-induced PA was evaluated. Compound **1** showed its greatest inhibitory effect at the concentration of 100 µg/mL, suppressing the PA with AA (0.0 ± 0.0%) and collagen (0.0 ± 0.0%) and decreasing PAF-induced PA (24.7 ± 2.9%) vs. control (86.3 ± 2.7%). However, the inhibitory effects of compound **1** were observed since the concentration of 50 µg/mL. Also, compound **2** reached its highest effect at 100 µg/mL, suppressing the AA- and collagen-induced PA (0.0 ± 0.0%) and decreasing those with thrombin (89.3 ± 1.9% vs. 41.0 ± 9.5%) and PAF (86.3 ± 2.7% vs. 24.7 ± 2.9%). Unlike compound **1**, the inhibitory effect of compound **2** on PA was evidenced since the concentration was 20 µg/mL. Finally, acetylated moscatilin (100 µg/mL) showed its greatest effect by suppressing the AA-induced PA (0.0 ± 0.0%) and decreasing that induced with collagen (86.4 ± 3.6% vs. 12.8 ± 6.3%) and PAF (86.3 ± 2.7% vs. 36.0 ± 7.8%). Nevertheless, we highlight that compound 3 had an inhibitory effect on the AA-induced PA since the concentrations of 5 and 10 µg/mL.

According to the above, the three compounds act as antiplatelets, showing a common effect on the AA-induced PA; however, compound **3** had the best effect with an inhibitory concentration 50 (IC_50_) of 11.2 µg/mL in comparison to compounds **1** (IC_50_: 61.8 µg/mL) and **2** (IC_50_: 37.2 µg/mL).

### 2.2. Dendrobium densiflorum

*D. densiflorum* has compounds that inhibit platelet activity. In 2001, Fan et al. [29] chemically extracted *D. densiflorum* stem using CH_2_Cl_2_. The extract was subjected to column chromatographic analysis and filtration, obtaining eighteen compounds. In two of these, their chemical structure was elucidated (densiflorol A and densiflorol B), and in a third one previously identified in the orchid, its chemical structure was confirmed (dendroflorin). However, the most relevant finding from the point of view of thrombosis was that compounds **6** (gigantol), **7** (moscatilin), **10** (homoeriodictyol), **14** (scoparone), and **15** (scopoletin) inhibited ADP (4 µM)-induced PA. For these tests, blood anticoagulated with sodium citrate (9:1, *v*/*v*) was obtained from the vena cava of male rats (Sprague Dawley strain; 250–300 g). PA was measured using the light transmission platelet aggregometry method. The inhibitory effect of the compounds was evaluated at concentrations of 50 and 100 µM, with percentages of inhibition that ranged from 5 to 29% and from 21 to 64% for the low and high concentrations, respectively. Briefly, at the concentration of 50 μM for compounds **7**, **10**, **14**, and **15**, PA with ADP (4 μM) was 30 ± 4%, 35 ± 1%, 29 ± 19%, and 40 ± 8%, respectively. These values were lower than 0.5% DMSO (42 ± 5%), the vehicle control. At the concentration of 100 µM for compounds **6**, **7**, **10**, **14**, and **15**, the ADP-induced PA was 29 ± 10%, 27 ± 8%, 21 ± 8%, 15 ± 11%, and 33 ± 5%, respectively. All percentages of PA were lower than the vehicle control (42 ± 5%). Regarding the percentage of inhibition, at the concentration of 50 μM, the compounds **7**, **10**, **14**, and **15** inhibited the ADP-induced PA in 29%, 17%, 31% and 5%, respectively. These values were lower than the control (42 ± 5%). At 100 µM, the percentage of inhibition was 31%, 36%, 50%, 64%, and 21% for compounds **6**, **7**, **10**, **14**, and **15**, respectively. Because compound **14** had previously been reported as a potent antiplatelet agent, it was used as a positive control. It was the compound with the highest percentage of inhibition, both at the concentration of 50 µM (31%) and 100 µM (64%). The sum of all the results suggests that *D. densiflorum* is a source of antiplatelet compounds.

### 2.3. Dendrobium longicornu

This orchid is a more abundant species in southwestern China. In 2008, a chemical investigation allowed the isolation, structural elucidation, and evaluation of the anti-platelet aggregation activity of new compounds of *D. longicornu* [30]. Additionally, other known compounds were identified. The stems of the orchid were collected from Xiaoshao in Kunming in Yunnan Province, P. R. China. Briefly, the air-dried stems of the plant were powdered and extracted with 95% aqueous EtOH (18 L × 3) under reflux. The EtOH extract was evaporated under reduced pressure and fractionated successively into CHCl_3_ and n-BuOH soluble fractions. Subsequently, using chromatography columns allowed the obtaining of five fractions of CHCl_3_ (A–E) and four fractions of n-BuOH (I–IV). The use of silica gel column chromatography and Sephadex LH-20 led to the purification of nineteen compounds. Fourteen of these were known: batatasin, gigantol, moscatilin, aloifol I, tristin, 3,3′,4-trihydroxybibenzyl, (3S,4S,5R)-3,4,5-trihydroxy-1-cyclohexenecarboxylic acid, naringenin, methyl β-orsellinate, episyringaresinol, episyringaresinol 4′′-O-β-D-glucopyranoside, 3-(3-methoxy,4-hydroxyphenyl)-1-propanol, 9-β-D-ribofuranosyl-9H-purin-6-amine, and eugenyl O-β-D-glucopyranoside. However, the compounds **1** (Longicornuol A), **2** (4-[2-(3-Hydroxyphenol)-1-methoxyethyl]-2,6-dimethoxyphenol), **3** (5-Hydroxy-7-methoxy-9,10-dihydrophenanthrene-1,4-dione), **4** (2,5,7-Trihydroxy-4-methoxy-9,10-dihydrophenanthrene), and **5** (Erythro-1-(4-O-β-D-Glucopyranosyl-3-methoxyphenyl)-2-[4-(3-hydroxypropyl)-2,6-dimethoxyphenoxy]-1,3-propanediol) were identified for the first time in the studied fractions III, C, C, II, and IV, respectively. For PA assays, the authors used PRP from New Zealand white rabbits and the samples were analyzed in the aggregometer (SHANDA PA-196). One milliliter of diluted blood containing 100 μL Chrono-Lume reagent (Chrono-Log Co., Havertown, PA, USA) and 20 μL testing sample was placed in a glass cuvette and subsequently incubated in the aggregometer at 37 °C for 5 min. ADP (8.93 µM) was used to induce PA, and ticlopidine was the positive control. Each aggregation assay was recorded until the maximal extent of aggregation was reached. The ATP release from the blood platelets was calculated based on ATP standards. The results showed that ticlopidine inhibited completely (100%) the ADP-induced PA at the concentration of 1088.4 µM, while the percentages of inhibition for compounds **1** (1525.6 µM), **2** (2348.7 µM), **3** (2789.1 µM), **4** (2767.4 µM) and **5** (1252.6 µM), were 73.6, 83.5, 83.3, 63.2 and 33.2%, respectively. These results suggest that the five compounds from *D. longicornu* have antiplatelet activity. However, their inhibitory effect was reached at higher concentrations than the positive control, so the authors concluded that compounds **1**–**5** exhibited weak anti-platelet activity.

### 2.4. Dendrobium trigonopus

*D. trigonopus* is distributed in southwest China. In 2008, Hu et al. [31] isolated nine compounds from this orchid, and among these the chemical structure of two new bibenzyls were elucidated. In addition, they reported the antiplatelet effect of one of the two new compounds. Briefly, orchid stems were separated, air-dried, and pulverized before extraction with 95% aqueous ethanol (15 L × 3) at reflux. The EtOH extract was successively fractionated into EtOAc and n-BuOH soluble partitions and subjected to column chromatography. Seven known isolated compounds were obtained from the six fractions (I–VI) derived from EtOAc. Five other fractions (a–e) were obtained from fraction II, where fraction c was purified by column chromatography [Sephadex LH-20, CHCl_3_/MeOH (1:1, *v*/*v*)] to obtain compounds **5** and **6**. Compound **7** was obtained from fraction IV. Compounds **3**, **4**, and **8** were obtained from fraction III, which was treated in the same way as fraction II, and compound **9** was obtained from fraction V. On the other hand, five fractions (A–E) were derived from the n-BuOH fraction, where fraction A was separated and subsequently purified by column chromatography (Sephadex LH-20, MeOH) to obtain compound **1** and **2**. Both were structurally elucidated with spectroscopic methods and named as trigonopols A and B. Next, in vitro evaluation of antiplatelet activity was performed on plasma from adult New Zealand white rabbits. To obtain PRP, citrated blood (3.8% trisodium citrate) was centrifuged at 1000 rpm for 10 min. Then, 20 µL of the test sample and 1 mL of diluted blood containing 100 µL of Chrono-Lume reagent were placed in a glass cuvette and incubated in an aggregometer (SHANDA PA-196) at 37 °C for 5 min. Ten microliters of ADP (250 µM) was used as platelet agonist. The degree of aggregation was determined from the maximum response height in Ohms, and the aggregation rate was established from the slope of the highest part of the curve. Ticlopidine (1.088 × 10^−3^ M) was used as a positive control showing 100% inhibition of PA. On the other hand, trigonopol A (1.434 × 10^−3^ M) inhibited 67.6% of PA; however, ticlopidine inhibited 100% of PA at a lower concentration (1.434 × 10^−3^ M). Therefore, the results suggest that trigonopol A exerts a weak anti-platelet activity.

### 2.5. Dendrobium venustum

The study [32] had the interest to evaluate the inhibitory effect of lusianthridin (a phenanthrene derivative isolated from *D. venustum*) on human PA. However, the study included other assays to investigate the possible mechanisms of inhibition. Briefly, the study was performed using human platelets from healthy volunteers. For the PA assay, PRP and PPP were obtained after centrifuging citrated blood samples at 21 °C, 200× *g* for 10 min, and 21 °C, 1500× *g* for 15 min, respectively. The agonists ADP (4 µM), collagen (2 µg/mL), and AA (0.5 µM) were used to induce PA. The PRP was pre-incubated with lusianthridin for 5 min at 37 °C. DMSO (0.5%) was considered the vehicle control and acetylsalicylic acid (ASA; 0.1 mM) the positive control. For AA-induced PA, the concentration range of lusianthridin was 0.0125 to 0.1 mM and 0.05 to 0.4 mM for ADP and collagen. The other assays performed in the study were molecular docking, cyclooxygenase activity, and the measurement of cAMP levels. According to the aggregation curves, the lusianthridin more selectively inhibited the second phase of ADP-induced PA compared with the first phase. PA was inhibited with the four concentrations of the compound (0.05, 0.1, 0.2, and 0.4 µM), with values of PA (%) close to the positive control (35% approximately) with a concentration of 0.2 µM. For AA-induced PA, lusianthridin at 0.0125 mM significantly increased the delaying time compared with the control group (80.0 ± 8.9 vs. 46.3 ± 5.6 s, respectively, *p* < 0.05). Regarding the percentage of PA, the compound tested at 0.025, 0.05, and 0.1 mM showed a similar inhibitory effect to the positive control (PA: 10% approximately). For PA assays induced with collagen, the authors found that lusianthridin at 0.4 mM concentration could significantly prolong the lag time of collagen-induced PA compared with vehicle control (114.5 ± 15.8 vs. 68.3 ± 5.0 s, respectively). At this concentration (0.4 mM), the percentage of PA was very similar to the values obtained for the positive control (15%, approximately). After calculating the percentage of inhibition of PA, the IC_50_ of lusianthridin for AA-, collagen- and ADP-induced PA were 0.02 ± 0.001 mM, 0.14 ± 0.018 mM, and 0.22 ± 0.046 mM, respectively. Therefore, the results suggest that platelets were more sensitive to the effect of the phenanthrene when PA was induced with AA. Among all the articles included in the review, the present one is the only one in which the mechanism of action was explored, so we dedicated some words to inform about the findings. The docking analysis showed that lusianthridin bound the cyclooxigenase-1 (COX-1) enzyme with a pi-donor hydrogen bond with Tyr-355, two hydrophobic bonds with Val-116, and Ile-89, and a hydrophobic bond with Leu-93. Arg-120 and Glu-524 surrounded the binding site of lusianthridin. Additionally, the binding affinity of lusianthridin was −7.2 kcal/mol. On the other hand, lusianthridin bound the cyclooxigenase-2 (COX-2) enzyme with two hydrogen bonds with Arg-44 and Asn-39 and three hydrogen bonds with Pro-153, Leu-152, and Cys-47. In addition, its binding site existed near the amino acid residues Glu-465 and Arg-469. Lusianthridin showed a binding affinity of −9.3 kcal/mol. The authors also found that the binding site of lusianthridin to COX-1 was partially similar to that of AA on the COX-1 enzyme, but it was far from that of AA on COX-2. Regarding the affinity for the cyclooxygenases, the binding affinity of lusianthridin was comparable to that of AA (−7.2 and −7.9 kcal/mol, respectively), but it was higher for lusianthridin (−9.3 kcal/mol) than AA (−7.3 kcal/mol) on COX-2. When the authors evaluated the effects of lusianthridin on cyclooxygenase enzymes, they found concentration-dependent inhibitory effects on both COX-1 and COX-2 enzymatic activities. For COX-1 assays, lusianthridin was evaluated at 6.25, 12.5, 25.0, 50, and 100 µM and at 0.125, 1.25, 12.5, and 125 µM for COX-2 assays. The IC_50_ values of lusianthridin on COX-1 and COX-2 were 10.8 ± 1.1 and 0.2 ± 1.6 μM, respectively. Finally, the authors evaluated the effects of lusianthridin on cAMP levels. It was found that ADP 4 μM significantly decreased the cAMP level in platelets (7.0 ± 0.2 pmol/10^8^ platelets) compared to the basal cAMP level (11.0 ± 0.4 pmol/10^8^ platelets). However, lusianthridin (0.4 mM) prevented the decrease in ADP-induced cAMP level (12.2 ± 1.4 pmol/10^8^ platelets). This effect was similar to that found with the positive control, 3-isobutyl-1-methylxanthine (11.7 ± 2.0 pmol/10^8^ platelets). In summary, this study shows that lusianthridin acts as an antiplatelet compound, mainly inhibiting the AA-induced PA by a probable mechanism that implies the inhibition of COX-1 and COX-2 enzymes, affecting the AA–thromboxane and adenylate cyclase pathways.

### 2.6. Cleisomeria lanatum

In 2022, the phytochemical, antioxidant, anti-inflammatory, and thrombolytic properties of *C. lanatum* were studied [33]. Orchids were collected in Cox’s Bazar, Chittagong, Bangladesh. The biological evaluation and chemical analysis of the composition of the methanolic extract (50 g/100 mL of methanol) were performed using the leaves, roots, and stems of *C. lanatum*. A qualitative and quantitative chemical analysis was carried out. Briefly, to evaluate the thrombolytic activity of the extract, venous blood from healthy volunteers was transferred to pre-weighed sterile microvials and incubated at 37 °C for 45 min. The serum was removed from each vial after clot formation, and the vials were weighed again to determine the clot weight. Subsequently, 100 μL of the extract was added to each vial and incubated at 37 °C for 90 min. After incubation, the liquid obtained in each vial was removed to weigh again. The difference obtained in the weight taken before and after adding the extract (50, 100, 150, 200, and 250 µg/mL) was expressed as a percentage of clot lysis; additionally, the IC_50_ was calculated. Streptokinase and water were used as positive and negative controls, respectively. The qualitative analysis results showed that the extracts from the three orchid tissues contain, to different degrees, alkaloids, flavonoids, saponins, tannins, phenols, terpenoids, steroids, glycosides, cardiac glycosides, quinine, coumarin, proteins, and resins.

Meanwhile, the quantitative analysis highlighted that the alkaloids had the highest concentration in root extract (106 mg/g), flavonoids (17.3 mg/g), phenols (179.7 mg/g), and tannins in stems extract (73.7 mg/g), and the highest protein content was found in leaf extract (0.133 mg/g). On the other hand, the ex vivo evaluation of thrombolytic activity revealed that extracts from the three orchid tissues were able to lyse the clots. The three extracts showed their greatest effect at the concentration of 250 µg/mL, but the highest one was for the stem extract with a percentage of clot lysis activity of 47.2%, while this value was 43.4% and 32.5%, for leaf and root extract, respectively. These data correlate with the IC_50_ of stem extract (163.8 µg/mL), which was lower than those found for leaf (IC_50_: 173.7 µg/mL) and root (IC_50_: 190.9 µg/mL) extract. The study clearly shows that *C. lanatum* is a source of thrombolytic compounds, mainly those contained in the stems with the highest concentration of phenols, flavonoids, and tannins.

### 2.7. Ephemerantha lonchophylla

Considering that the hydroethanolic extract of *E. lonchophylla* stems has an inhibitory effect on PA with AA, Chen et al., in 2000 [34], performed a chromatography analysis using silica gel and Sephadex LH-20 columns to evaluate the antiplatelet activity of several fractions. The orchid stems were obtained from a local market in Taipei, Taiwan. The extraction used 3 kg of orchid stems (10 L × 3). The specific conditions for the chromatography analysis can be consulted in the article. Ten fractions were obtained from the extract, and four compounds were identified by comparing their spectral data (EI-MS and MNR) with those published: **1** (denbiobin), **2** (3,7-dihydroxy-2,4-dimethoxyphenanthrene), **3** (3-methylgigantol), and **4** (erianthridin). Rabbit platelets were used to explore the inhibitory effects of compounds on thrombin (0.1 U/mL)-, AA (100 µM)-, collagen (10 µg/mL)-, and PAF (2 ng/mL)-induced PA. The light transmission platelet aggregometry method was used to explore the antiplatelet effects of compounds. For compound **1**, one concentration was probed (100 µg/mL), with six concentrations for compound **2** (2, 5, 10, 20, 50, and 100 µg/mL), and five concentrations for compounds **3** (5, 10, 20, 50 and 100 µg/mL) and **4** (1, 2, 5, 10 and 100 µg/mL). ASA (50 µM) was the positive control. PA assays revealed the antiplatelet activity of compounds **1**–**4**. Compound 1 decreased PA induced by thrombin (87.6 ± 1.2% vs. 92.6 ± 1.4%), AA (71.6 ± 2.2% vs. 85.9 ± 0.5%), and collagen (80.9 ± 2.6% vs. 88.1 ± 0.9%) vs. control (0.5% DMSO). Meanwhile, compounds **2**–**4** showed the greatest inhibitory effect at 100 µg/mL concentration. Briefly, compound **2** suppressed the AA-induced PA (0.0 ± 0.0%) and decreased that induced with collagen (11.8 ± 1.7% vs. 88.1 ± 0.9%) and PAF (7.0 ± 2.8% vs. 88.1 ± 0.7%). A similar profile was seen for compound **3** because it suppressed the AA-induced PA (0.0 ± 0.0%) and decreased that induced with collagen (4.2 ± 2.0% vs. 88.1 ± 0.9%) and PAF (1.4 ± 1.2% vs. 88.1 ± 0.7%) vs. control. Unlike compounds **1**–**3**, compound **4** (100 µg/mL) inhibited PA with the four agonists. It suppressed the AA-induced PA (0.0 ± 0.0%) and decreased that induced with thrombin (86.3 ± 0.4% vs. 92.6 ± 1.4%), collagen (16.3 ± 1.8% vs. 88.1 ± 0.9%), and PAF (34.2 ± 5.1% vs. 88.1 ± 0.7%). According to the above, the four compounds mainly inhibit the AA activation pathway in platelets; however, compound **4** (erianthridin) had the most powerful antiplatelet effect (IC_50_: 9 µM) in comparison to compounds **2** (IC_50_: 24 µM) and **3** (IC_50_: 30 µM). We also highlight that compounds **4** and **2** suppressed AA-induced PA at a lower concentration (10 and 20 µg/mL, respectively) than the positive control (50 µg/mL). This research shows that *E. lonchophylla* is a source of antiplatelet compounds.

### 2.8. Calanthe arisanensis

*C. arisanensis* is an endemic orchid from Taiwan. In 2013, the methanolic extract of *C. arisanensis* leaves was partitioned with ethylic acetate and water to obtain the ethylic acetate fraction [35], which was subjected to silica CC eluting with CHCl_3_-MeOH. After removing a large amount of chlorophyll and fatty acids, eight compounds were isolated: three phenanthrenes (compounds **1**–**3**): **1** (2,7-dihydroxy-3,5-dimethoxy-9,10-dihydro-phenanthrene), **2** (2,7-dihydroxy-4-methoxy-9,10-dihydro-phenanthrene), **3** (densiflorol B); four indole alkaloids (compounds **4**–**7**): **4** (tryptanthrin), **5** (indirubin), **6** (isatin), **7** (indican); and one steroid: compound **8** ((24R)-4α,24-dimethyl-5α-cholesta-7,22-dien-3β-ol). Compounds **3**–**8** were found for the first time in the leaves of *C. arisanensis*; however, only phenanthrene 1 and 2 had a potent inhibitory effect on the collagen-induced (10.0 µg/mL) PA, with IC_50_ values of 0.2 and 1.3 µg/mL, respectively. These values were lower than the positive control (ASA) with an IC_50_ of 13.8 µg/mL. Considering the antiplatelet activity of phenanthrenes 1 and 2, eleven naturally occurring phenanthrenes previously identified in the orchid were isolated from the roots of *C. arisanensis*, and another eighteen phenanthrenes were synthesized to evaluate their effects on collagen- and thrombin-induced PA. Among natural phenanthrenes, compounds **9** (IC_50_: 10.7 µg/mL), **11** (IC_50_: 3.4 µg/mL), **12** (IC_50_: 8.9 µg/mL), **13** (IC_50_: 4.0 µg/mL), **14** (IC_50_: 1.8 µg/mL), **15** (IC_50_: 2.6 µg/mL), and **16** (IC_50_: 2.4 µg/mL) showed more potent inhibition against PA induced with collagen, with IC_50_ values lower than the positive control (IC_50_: 13.8 µg/mL). Meanwhile, the natural phenanthrenes did not have an essential effect on thrombin-induced (0.05 U/mL) PA. On the other hand, phenanthrenes 19 (IC_50_: 3.6 µg/mL), 20 (IC_50_: 0.2 µg/mL), 21 (IC_50_: 0.2 µg/mL), 22 (IC_50_: 0.1 µg/mL), 29 (IC_50_: 1.9 µg/mL), 30 (IC_50_: 3.0 µg/mL), 31 (IC_50_: 3.8 µg/mL), 32 (IC_50_: 0.8 µg/mL), 33 (IC_50_: 2.4 µg/mL), 34 (IC_50_: 1.0 µg/mL), and 35 (IC_50_: 0.2 µg/mL) showed a more potent inhibitory effect on the collagen-induced PA than the positive control (IC_50_: 8.9 µg/mL). For PA with thrombin, the molecules 20, 21, and 22 showed the best inhibitory effect, with IC_50_ values of 0.8, 1.0, and 1.1 µg/mL. The study informs about the usefulness of phenanthrenes as new antiplatelet agents. The original article should be read to know about the chemical structures of the phenanthrenes with antiplatelet activity.

### 2.9. Cephalanceropsis gracilis

In 2018, Sharma et al. [36] performed the ultrasound-assisted synthesis of Cephalandole A 2 (a compound isolated from *C. gracilis*) and other analogs derived from the same compound (a–o) to evaluate their biological activity, including antiplatelet activity. For this purpose, Cephalandole A 2 and its analogs (a–o) were dissolved in DMSO (0.5%) before testing to eliminate the effects of the solvent on PA. Rabbit blood extracted from the marginal vein of the ear was used and mixed with EDTA to a final concentration of 6 mM. PRP was obtained by centrifuging this mixture at 90× *g* for 10 min. Subsequently, the PRP was centrifuged for 10 min at 500× *g*. The platelet button obtained was then washed with Tyrode’s solution (Ca^+2^ free) containing 2 mM EDTA and 0.1 or 3.5 mg/mL bovine serum albumin and centrifuged at 500× *g* for 10 min. The platelets were then washed with EDTA-free Tyrode’s solution, which was centrifuged under the same conditions. Finally, the platelet button was suspended in a Tyrode solution of the following composition: NaCl (136.8 mM), KCl (2.8 mM), NaHCO_3_ (11.9 mM), MgCl_2_ (2.1 mM), NaH_2_PO_4_ (0.33 mM), CaCl_2_ (1.0 mM), and glucose (11.2 mM) containing bovine serum albumin (0.35%). Three minutes before adding the agonist, the platelet suspension was shaken at 1200 rpm. AA (100 µM) was used as a platelet agonist and ASA as the positive control. A turbidimetric method (Lumi-aggregometer) was used to measure PA. The percentage of aggregation was expressed as 0% aggregation for the absorbance of the platelet suspension and 100% aggregation for the absorbance of Tyrode’s solution without platelets. The inhibitory effect on AA-induced PA was reported as IC_50_ (µg/mL). The chemical structure of Cephalandole A 2 (2-oxo-benzo [1,4] oxazine) contains bicyclic rings A and B linked to C-3 of the indole rings C and D. In this regard, Cephalandole A 2 having no substitutions on the A or D rings had a greater inhibition of PA (IC_50_ = 19.3 ± 0.3 µg/mL) in comparison to the positive control (IC_50_ = 22.2 ± 0.5 µg/mL). Similarly, compound c, having the electron-withdrawing groups (NO_2_) at the C-6 position of ring A, has a higher antiplatelet activity (IC_50_ = 20.2 ± 0.3 µg/mL) than the positive control. Conversely, the introduction of the electron-donating groups (CH_3_) at the C-6 position of the A ring (g) resulted in the complete loss of the antiplatelet activity (IC_50_ = 111.8 ± 1.9 µg/mL). However, in compound h, introducing NO_2_ instead of CH_3_ (C-6 of ring A) restored the inhibitory effect (IC_50_ = 30.2 ± 0.7 µg/mL). Similarly, the introduction of NO_2_ at C-7 of ring A of compound i, maintained the inhibitory effect (IC_50_ = 21.6 ± 0.3 µg/mL), with a lower intensity than compound f (IC_50_ = 19.0 ± 0.2) but similar to that of the positive control (IC_50_ = 22.2 ± 0.5 µg/mL). On the other hand, substituting the electron-donating group OMe on the indole ring D (21 k) of Cephalandole A decreased the antiplatelet activity of this compound (IC_50_ = 52.5 ± 1.1 μg/mL). Similarly, substituting the Cl, Br, or CH_3_ group in the A ring of Cephalandole A did not improve the antiplatelet activity of compounds m-n, whereas substitution of O by N in the 2-oxo-benzo [1,4] oxazine moiety of the tested compound showed the lowest inhibitory activity on PA (IC_50_ = 104.5 ± 1.6 μg/mL). This indicated that the electron-withdrawing groups enhance the inhibitory activity of Cephalandole A on the AA-induced PA, and the electron-donating groups lead to decreased or total loss of this activity.

### 2.10. Gastrodia elata

Several studies have shown that *G. elata* has antithrombotic properties. In 1995 [37], the inhibitory effects of five fractions of the hydroethanolic extract of *G. elata* [hexane (I), methanol (II), ethyl acetate (III), butanol (IV) and water (V)] were evaluated on rat platelets. The light transmission platelet aggregometry method was used for this aim. Fractions II, III, and IV at 2.5 mg/mL suppressed the ADP- and collagen-induced PA. ASA (1 mg/mL) was the positive control. The antiplatelet effects of fractions II, III, and IV were also evaluated in mouse and rat thrombosis models. In mice, the bioactive fractions increased the percentage of animals recovered from thrombotic paralysis compared to the control group (17%). Briefly, the percentage of mice recovered was 40, 44, and 32% after an oral administration (100 mg/kg) of fractions II, III, and IV, respectively. Similarly, in rats, the oral administration of fractions II, III, and IV (500 mg/kg) reduced the effects of the intravenous administration of collagen (60 µg/10 mL saline/kg) on platelet count. For control, collagen decreased the platelet count from 650.3 ± 97.9 × 10^9^ cells/L (100%) to 551.3 ± 84.7 × 10^9^ cells/L (84.5 ± 3.8%), a reduction of 15.5%. For fractions II, III, and IV, the mean percent reduction in platelet count was 5.4% (710.5 ± 69.7 × 10^9^ cells/L to 673.5 ± 87.8 × 10^9^ cells/L), 13.7% (615.5 ± 63.2 × 10^9^ cells/L to 531.0 ± 57.9 × 10^9^ cells/L), and 10% (618.3 ± 132.0 × 10^9^ cells/L to 558.5 ± 131.4 × 10^9^ cells/L), respectively. Both in vitro and in vivo models suggest that *G. elata* contains antiplatelet compounds.

Eleven years later (2006) [38], the in vitro and in vivo effects of *G. elata* rhizome fractions on PA induced by ADP (3 µmol/L), AA (780 µmol/L), and PAF (15 nmol/L) were studied. PA was determined by the light transmission platelet aggregometry method. Anticoagulated blood (3.8% sodium citrate) from the carotid artery of rabbits was subjected to centrifugation to obtain platelet-rich plasma. The fraction G_2_ of *G. elata* rhizomes inhibited PA in both in vitro and in vivo models. In vitro, the fraction G_2_ (0.15, 0.30, 0.60, 1.2, and 2.4 mg/mL) decreased the ADP-induced PA from the concentration of 0.3 mg/mL (35.2 ± 7.7%) vs. control (49.7 ± 7.4%), showing its greatest effect at the concentration of 2.4 mg/mL (11.2 ± 6.5% vs. 49.7 ± 7.4%). Regarding the AA-induced PA, the fraction G_2_ had the inhibitory effect at the concentration of 2.4 mg/mL, with a percentage of PA lower (20.6 ± 19.8%) than the control (72.0 ± 9.5%). Meanwhile, the fraction G_2_ at concentrations of 1.2 and 2.4 mg/mL decreased PAF-induced PA compared to the control (1.2 mg/mL: 38.6 ± 14.3% vs. 66.9 ± 10.9%; 2.4 mg/mL: 6.2 ± 7.5% vs. 66.9 ± 10.9%). For in vitro results, the fraction G_2_ inhibited the ADP-induced (10 µmol/L) PA in rat platelets. Five groups of rats were made. Groups were administered (intragastrically) with saline solution (control group), ASA 100 mg/kg, or three different doses of the orchid fraction: 150, 200, and 250 mg/kg. The fraction was administered continuously for 5 days. ADP-induced PA was 41.1 ± 15.8%, 46.7 ± 10.8%, and 47.6 ± 15.0% at concentrations of 150, 200, and 250 mg/kg of fraction G_2_, respectively. These values were lower than the control group (61.5 ± 8.2%). Finally, the study evaluated the protective effect of fraction G_2_ on ADP-induced acute pulmonary embolism in mice. Mice (18 to 22 g) were used and divided into four groups: the control group, to which the vehicle was administered; the positive control group that was administered with 10 mg/kg of ASA; and the groups that were administered with 10 and 5 mg/kg of fraction G_2_. Treatments were administered (two times a day for 3 days) using a nasogastric tube for all groups. The results showed that both ASA and fraction G_2_ (10 and 5 mg/kg) decreased the percentage of dead mice due to thrombosis induced by ADP (300 mg/kg). ADP was administered intravenously. In the control group, 93.8% of mice died, a value that decreased after treatment with ASA (41.7%). However, the percentage of dead mice was lower with fraction G_2_, 26.7% and 15.4% at 5 and 10 mg/kg doses, respectively. The results show the in vitro and in vivo antiplatelet activity of the fraction G_2_ of *G. elata* rhizome.

Regarding the bioactive compounds of *G. elata*, in 2004 [39], 11 compounds (9 phenolic compounds and 2 furan-type compounds) were identified in the EtOAc and CHCl_3_ fractions of the methanolic extract of orchid tubers. The compounds were 1 (4,4’-dihydroxybenzylsulfone), 2 (4-dihydroxybenzylmethylether), 3 (4-dihydroxybenzylalcohol), 4 (4-dihydroxybenzaldehyde), 5 (4,4´-dihydroxybenzyl sulfoxide), 6 (gastrodin), 7 (4-[4′-(4”-hydroxybenzyloxy) benzyloxy]benzyl methyl ether), 8 (4,4′-dihydroxy-diphenyl methane), 9 (4,4-dihydroxy-dibenzylethe), 10 (5-hydroxymethyl-2-Furancarboxadehyde), and 11 (cirsiumaldehyde). The activity of the compounds was determined in rat PRP to perform PA induced by ADP (2–5 µM), collagen (2–5 µg/mL), epinephrine (1–4 µM), AA (10–40 µM), and thromboxane A2 mimetic agent (U46619: 1–5 µM). The eleven compounds showed inhibitory effects; however, their potency did not consistently exceed that observed with the positive control (ASA). Briefly, the IC_50_ of the control was 420, 53, 66, and 340 µM for the collagen-, epinephrine-, AA-, and U46619-induced PA, respectively. We highlight that compound **9** showed a more potent inhibitory effect on PA induced with collagen (IC_50_: 5 µM), epinephrine (IC_50_: 3 µM), and U46619 (IC_50_: 33 µM) vs. positive controls (IC_50_: 420, 53 and 340 µM, respectively). Compound 8 was the only one that showed a greater inhibitory effect (IC_50_: 30 µM) on AA-induced PA vs. positive control (IC_50_: 66 µM). The IC_50_ of compound **1** (83 µM) for PA with U46619 and IC_50_ of compound **7** (191 µM) for PA with collagen were also lower than the positive control (IC_50_: 340 µM and 420 µM, respectively). Therefore, the compound 4,4-dihydroxy-dibenzylethe was the most potent antiplatelet agent in *G. elata*.

In another study, Ding et al., in 2007 [40], evaluated the effects of polysaccharide 2-1 from *G. elata* (PGE2-1) on blood coagulation. The study used healthy mouse blood (20 ± 2 g) to measure CT (glass method) and BT (tail-cut method). For these tests, PGE2-1 (30, 60, and 120 mg/kg), saline solution (1 mL/kg: as a negative control), and heparin (50 U/kg: positive control) were administered intraperitoneally. BC (tail cut at 5 min) and RT were evaluated. The percentage of ADP-induced (0.1 mol/L) PA was determined to evaluate the effects of PGE2-1 on platelets, reporting the maximum PA and PA at 5 min, while PT, APTT, and TT informed about the effect of PGE2-1 on the plasma phase of hemostasis. The effects of PGE2-1 were also evaluated in an ADP-induced thrombosis model. For this experiment, PGE2-1 was administered to mice for 7 days, one hour after the last dose, and they were administered 250 mg/kg of ADP. The times when the mice had difficulty breathing, twitching when they lay down or were unable to move, and when they resumed autonomous activities were recorded. On the other hand, the effect of PGE2-1 on an in vitro model of thrombosis in rats was performed. Five groups of rats were formed (males and females alike) and were sublingually administered with saline (1 mg/kg), heparin (50 U/kg), or PGE2-1 (30, 60, 120 mg/kg). A precise description of the study can be found in the original publication. After the experiments, it was possible to determine the dry and wet weight of the thrombus. Also, the effect of PGE2-1 on arteriovenous shunt thrombosis in rats was evaluated. Readers will be able to review the assay details in the original article. The wet weight of the thrombus was obtained from these tests to calculate the thrombosis inhibition rate.

First, it was observed that the CT and BT of mice treated with PGE2-1 (60 and 120 mg/kg) were significantly prolonged compared to the negative control group (CT: 4.2 ± 0.2 min; BT: 9.9 ± 0.8 min). For CT, values of 6.3 ± 0.4 and 8.2 ± 0.3 min were obtained at concentrations of 60 and 120 mg/kg of PGE2-1, respectively. For BT, the values were 12.05 ± 1.5, 13.76 ± 1.91, and 16.51 ± 2.89 min for the three concentrations of PGE2-1 (30, 60 and 120 mg/kg), respectively. Similar data were observed after measuring BC and RT. The BC went from 0.1 ± 0.0 A_540_ in mice treated with saline to 0.4 ± 0.1 and 0.3 ± 0.1 in mice treated with 60 and 120 mg/kg of PGE2-1, respectively. Similarly, the RT of mice treated with PGE2-1 (30, 60, and 120 mg/kg) was longer. Compared to the control (6.1 ± 0.0 A_540_), the highest RT was observed in mice treated with 30 mg/kg of PGE2-1 (7.6 ± 0.3 A_540_). Despite the positive effects of PGE2-1 on CT, BT, BC, and RT, none of these were greater than those of heparin (positive control). On the other hand, PGE2-1 inhibited the ADP-induced PA, with the greatest inhibitory effect reached at the concentration of 120 mg/kg. In addition, PGE2-1 (10, 20, and 40 mg/mL) prolonged TT and APTT in human plasma but not PT. TT was 11.3 ± 0.8 s and 11.5 ± 1.2 s at concentrations of 10 and 20 mg/mL of PGE2-1, respectively. In both cases, the TT was more prolonged than the control (7.9 ± 0.6 s). Furthermore, APTT was also prolonged vs. control (33.2 ± 3.2 s), with values of 41.4 ± 2.1 s (PGE2-1: 20 mg/mL) and 45.1 ± 3.1 s (PGE2-1: 40 mg/mL) from the effect of the polysaccharide. However, PGE2-1 had a lower anticoagulant effect than heparin.

Then, the effects of PGE2-1 on PA and ADP-induced thrombosis in mice were evaluated. The study found that PGE2-1 shortened the time mice stopped showing symptoms of thrombosis and the time in which they resumed their daily activities. The effect was dose-dependent, with better results than heparin at 60 and 120 mg/kg of PGE2-1. In other words, the mice’s recovery time with heparin was 18.2 ± 1.7 min, but it was 16.6 ± 1.9 and 11.3 ± 1.0 min at PGE2-1 doses of 60 and 120 mg/kg, respectively.

For the in vitro rat thrombosis model, PGE2-1 decreased thrombus length and wet and dry weight. Briefly, the length, wet weight, and dry weight of thrombi in the untreated rats were 4.7 ± 0.8 cm, 230.6 ± 40.9 mg, and 56.8 ± 8.7 mg, respectively, while for thrombi in rats treated with PGE2-1 (120 mg/kg), the length (1.7 ± 0.4 cm), wet weight (130.7 ± 11.1 mg), and dry weight (22.0 ± 3.6 mg) were lower. Similar but less intense results were observed at PGE2-1 doses of 30 and 60 mg/kg. Additionally, the wet weights of the experimental arteriovenous thrombosis decreased from the effect of PGE2-1. For untreated animals, the wet weight of the thrombus was 88.5 ± 2.8 mg vs. 59.8 ± 2.7, 44.8 ± 3.8, and 34.1 ± 4.6 mg for rats treated with PGE2-1 at doses of 30, 60 and 120 mg/kg, respectively. These data correspond to a percentage of inhibition of 32.5%, 49.0%, and 61.5%. In summary, PGE2-1 has anticoagulant activity by prolonging APTT and TT tests and acts as an antiplatelet by inhibiting ADP-induced PA. In addition to the in vitro evidence, animal thrombosis models support the potential use of PGE2-1 in treating thrombosis.

In a different context, it was demonstrated that *G. elata* contains thrombolytic compounds [41]. The study is based on using centrifugal partition chromatography (CPC), a liquid–liquid partition technique that does not use a solid matrix. With this technique and the use of preparative high-performance liquid chromatography (HPLC), the study aimed to purify phenolic compounds with plasmin-stimulating activity. Briefly, *G. elata* rhizomes were purchased from an oriental herb market in Kyungdong, Seoul, Korea. A methanolic extraction was performed by ultrasound. *G. elata* rhizomes (1.8 kg) were ground into powder and extracted thrice with methanol (3 L) for 90 min. Ninety-six grams of the methanolic extract were suspended in 1 L of water and then partitioned with n-hexane, ethyl acetate, and n-butanol (1 L × 3 times), obtaining 2.07, 2.7, and 9.42 g of the n-hexane, ethyl acetate, and n-butanol extracts, respectively. The in vitro activity of plasmin was evaluated using a commercial test based on the quantification of the AFC fluorophore released from a fluorogenic substrate by the action of plasmin. The experimental conditions for CPC and preparative HPLC, as well as compound identification by ESI-MS and NRM, can be consulted in the original article. The study found that the ethyl acetate fraction and water increased plasmin activity. By contrast, n-hexane and n-butanol fractions inhibited the enzyme. Considering these results, new experiments were performed with ethyl acetate and water fractions at different concentrations (5, 50, 500, and 5000 µg/mL). It was found that both fractions increased plasmin activity; however, a concentration-dependent response was only observed for the aqueous fraction. Subsequently, the compounds with probable thrombolytic activity were isolated and purified using the abovementioned techniques. From the fraction of ethyl acetate, five fractions (I–V) were obtained and six compounds were identified: **1** (4-Hydroxybenzyl alcohol), **2** (4-Hydroxybenzoic acid), **3** (4-Hydroxybenzaldehyde), **4** (4-Ethoxymethyl phenol), **5** (4,4′-Oxybis(methylene)diphenol), and **6** (4,4′-Methylenediphenol). In addition, two compounds were purified from the aqueous fraction: Parishin and Parishin B. The stimulatory effect of the purified compounds on plasmin activity was evaluated at the concentration of 1 μM. Compared to the control (50 nM plasmin), compounds **1**, **3**, and **4** stimulated plasmin activity; however, compound **1** stimulated it more than double. In conclusion, *G. elata* contains phenolic compounds with fibrinolytic activity.

### 2.11. Laelia furfuracea

In 2022 [42], our working group investigated the anticoagulant effects of *L. furfuracea*, an endemic orchid from Mexico. Specifically, the effect of the foliar hydroethanolic extract (5–60 mg/mL) and its fractions (1–10 mg/mL: aqueous, butanol, ethyl acetate, chloroform, and hexane) was evaluated. The study was performed ex vivo using platelet-poor plasma (PPP) from untreated patients with venous thrombosis. A hydroethanolic extraction was carried out using the Soxhlet method, and then the extract was partitioned using solvents of different polarities. The effect of the extracts and fractions on secondary hemostasis was evaluated by measuring the PT, TT, and APTT. At the same time, the possible bioactive compounds were identified in an ultra-high-performance liquid chromatographic system (UPLC, Thermo Scientific, Waltham, MA, USA, Ultimate 3000) coupled to an Impact II mass spectrometer (Bruker Daltonics, Billerica, MA, USA) equipped with electrospray ionization and quadrupole with time of flight (UPLC-ESI-Qtof). The results showed that the hydroethanolic extract of *L. furfuracea* leaves contains anticoagulant compounds because it prolongs the three coagulation times in a concentration-dependent manner (5–60 mg/mL). Briefly, the extract at a concentration of 25 mg/mL of PPP prolonged the PT and TT by 49.1 ± 0.8 s and 30.1 ± 0.9 s, respectively, compared to basal determinations (without extract). For the APTT, the prolongation was 61.8 ± 3.4 s at the concentration of 15 mg/mL of the extract. Regarding the identification of the compounds, the qualitative analysis tentatively identified 3-methylxanthine, malic acid, isocitric acid, succinic acid, O-methyl-D-glucopyranose, protocatechuic acid, rosmarinic acid, tryptophan, syringic acid acetate, luteolin-7,3 ‘-di-O-glucoside, kaempferol-3-O-rutinoside, kaempferol-7-O-glucoside, apigetrin, (+)-pinoresinol 4-glucoside, N-acetyl-L-glutamine, and N-acetylarginine. This study did not explore the anticoagulant activity of these compounds, so it is still unknown what the bioactive compounds are. When the extract was fractionated, the anticoagulant effect on some fractions increased. Of the five fractions, the ethyl acetate fraction showed the greatest effect. It prolonged the clotting time by more than 300 s at 30 mg/mL for TT, 28 mg/mL for PT, and 15 mg/mL for APTT. Prolonging the three coagulation times made us think about a likely thrombin inhibition. However, when the anticoagulant effect of the extract was challenged by increasing the thrombin concentration (2 to 40 IU/mL) in the TT assay, the excess enzyme failed to neutralize the anticoagulant effect.

Table 1 summarizes the main results of the orchids’ antiplatelet, anticoagulant, and fibrinolytic activity.

## 3. Discussion

To understand antithrombotic strategies, it is imperative to know that venous and arterial thrombi are different. The hemodynamic factors of blood vessels determine that a thrombus in the artery is composed mainly of platelets and a discrete fibrin mesh, while in a vein, fibrin is the most abundant component of a thrombus that encompasses many erythrocytes and few platelets [11]. Thus, anticoagulants are frequent in patients with venous thrombosis [43] and antiplatelet agents in patients with arterial thrombosis; however, sometimes, either both are used simultaneously [44] or thrombolytic (fibrinolytic) drugs are added to the treatment of venous and arterial thrombosis [45]. Hence, the study of natural products with antithrombotic activity must include searching for anticoagulant, antiplatelet, and thrombolytic compounds.

The most used anticoagulants worldwide are vitamin K antagonists (VKAs) and heparins (unfractionated and low molecular weight). VKAs inhibit the reduction in vitamin K in the liver, affecting the carboxylation of factors II, VII, IX, and X. Meanwhile, heparins potentiate the activity of antithrombin, a natural anticoagulant in humans that mainly inhibits activated factors II and X [46]. VKAs and heparins are indirect anticoagulants as they do not exert direct inhibitory effects on coagulation factors. However, today a new generation of anticoagulants is available, drugs with the ability to directly inhibit activated factors II and X [46]. In the case of antiplatelets, ASA and clopidogrel are the most used drugs. ASA acts inhibiting the platelet enzyme cyclooxygenase, affecting the synthesis of thromboxane A_2_, while clopidogrel inhibits platelet ADP receptors [47]. Therefore, these drugs inhibit PA. Unlike anticoagulants and antiplatelets that prevent thrombus formation, fibrinolytic drugs act directly on the thrombus. These include recombinant proteins of tissue plasminogen activator, the enzyme urokinase produced by renal parenchyma cells, and streptokinase, a protein synthesized by β-hemolytic streptococcus [45]. In any case, fibrinolytics promote the activation of plasminogen into plasmin to lyse the fibrin mesh.

The benefit of current antithrombotic treatment is unquestionable; even so, many research groups worldwide seek to offer better thrombosis treatments by studying natural compounds from plants. In the case of orchids, information on antithrombotic properties may be scarce if it considers the immensity of species that comprise this family [18]. Despite this, the findings summarized in this review suggest that orchids are an important source of antithrombotic molecules.

The antiplatelet effect was evident in nine orchids. For *D. loddigesii*, *D. densiflorum*, *D. longicornu*, *D. trigonopus*, *D. venustum*, *E. lonchophylla*, *C. arisanensis*, and *C. gracilis*, the effect was evaluated in vitro [28,29,30,31,32,34,35,36]. Instead, the antiplatelet effect of *G. elata* was studied in vitro and in vivo [37,38,39]. Of course, the use of animal models to explore the impact of *G. elata* on platelets is one step further compared to the other orchids [48]. Several compounds with antiplatelet activity in vitro were identified (Table 1). These included phenols, furans, terpenes, and phenanthrenes, among other compounds. For many years, many in vitro, in vivo, and clinical studies have documented the effects of phenolic compounds on cardiovascular diseases and PA [49], so finding these compounds in the orchids with antiplatelet activity was expected. On the contrary, we consider it relevant that different phenanthrenes and PGE2-1 act as natural antiplatelet agents, allowing new studies to identify these compounds in other orchid species to evaluate their inhibitory properties. We highlight that PGE2-1 was the only orchid compound in which the antiplatelet activity was demonstrated in vivo [40].

On the other hand, the anticoagulant activity of orchids was explored only in *G. elata* [40] and *L. furfuracea* [42]. The evidence is derived from in vitro studies for both orchids, with a more potent apparent effect for PGE2-1 of *G. elata* on the APTT and TT [40] compared to the hydroethanolic extract of *L. furfuracea leaves* and its fractions [42]. This is an expected result because the study of *G. elata* comprised a purified compound, but the relevance of this orchid does not lie in this comparison; what is relevant for this review is that PGE2-1 is a dual inhibitor of hemostasis, acting as an anticoagulant and antiplatelet compound. This could become very relevant for the treatment of thrombosis because the anticoagulant and antiplatelet effects might benefit patients with acute coronary syndrome, atrial fibrillation, or stroke, in whom the combined use of anticoagulants and antiplatelet drugs may be simultaneously indicated [50].

In addition, in 2006 [51], the anticoagulant activity of gastrodin was studied. Interestingly, this compound prolonged the clotting time but did not affect kaolin-activated partial thromboplastin time and PT. A docking analysis showed that gastrodin binds strongly to the “a and b” polymerization sites of fibrinogen, thereby interfering with fibrin polymerization [51]. Considering these findings, it is likely that the gastrodin identified in *G. elata* exerts dual antithrombotic activity through inhibition of PA and fibrin generation.

This review also reports orchids with fibrinolytic compounds. Specifically, it informs that *C. lanatum* [33] and *G. elata* [41] exert this activity. For *C. lanatum*, the clot lysis was evaluated, while in *G. elata*, the authors employed an assay to measure plasmin activity. Due to methodological differences, the results of both studies were not compared to determine which orchid had the best thrombolytic effect. Despite this, an outstanding fact about *G. elata* is that it contains compounds with the three desired effects of antithrombotic treatment (anticoagulant, antiplatelet, and fibrinolytic). Inevitably, this evidence and the beneficial effects observed in the in vivo thrombosis model position this orchid as an important plant source for developing new antithrombotic treatments.

Although evidence on orchids shows the presence of antithrombotic compounds, some gaps should be addressed in further studies. First, only one study explored the mechanism of action of antiplatelet compounds [32]. For the rest of the studies, it is unknown what the platelet targets for the orchid compounds are; however, in all cases, regardless of the agonist used, we can assume an overall mechanism, inhibition of platelet receptor α_IIb_β_3_ activation, because PA is mediated by this receptor [3]. The precise mechanism of PA inhibition for each identified compound remains to be elucidated in further studies. This is important because the effectiveness of an antiplatelet agent may depend not only on the potency of its inhibitory effect but also on inhibiting a specific target on platelets [52]. On the other hand, a possible anticoagulant mechanism for *G. elata* derives from the results of clotting times. The prolongation of APTT and TT but not PT suggests that thrombin is the target of PGE2-1 because APTT and TT are more sensitive to thrombin inhibition than PT [53,54]. Of course, this is an assumption, and the evaluation of the amidolytic activity of thrombin in the presence of PGE2-1 may clarify the proposed mechanism [55]. For *L. furfuracea* [42], suggesting a mechanism of action would be inappropriate because anticoagulant compounds were not identified. Therefore, the next step in this research should be the identification of these compounds; only then could it be possible to determine the potential of the orchid in the field of anticoagulants. Regarding the fibrinolytic effect of *G. elata* [41], we believe that orchid compounds enhance the enzymatic activity of plasmin because the use of exogen plasmin and a chromogenic substrate for this enzyme may limit the possibility of other mechanisms. A molecular docking analysis would help to understand the mechanism by predicting the binding affinity of the compounds to plasmin [56]. For *C. Lanatum*, compounds with fibrinolytic activity are unknown, so there is no mechanism to propose. Further studies are necessary to identify which phenols, flavonoids, and tannins mainly contained in the stems of the orchid have fibrinolytic activity. Second, there is scarce evidence in vivo about the antithrombotic activity of orchids; inevitably, in vitro findings may not be reproduced in vivo. In addition, we highlight that the evidence of antiplatelet effects in vitro was mainly obtained from animal platelets, whereas those from humans are easy to obtain. Indeed, this is not a limitation and does not affect the quality of the research. However, we must have in mind the possible differences in terms of hemostasis between animal species [57]. Third, neither the study with *G. elata* [40] nor that with *L. furfuracea* [42] evaluated the in vitro anticoagulant effects beyond clotting times. These traditional coagulation tests were developed while discovering the coagulation cascade more than fifty years ago and contribute to the current understanding; however, these tests may not reflect the complexity of blood coagulation. In addition, these tests only inform on the initiation of coagulation but not the whole hemostatic capacity in terms of clot formation and thrombin generation [58]. Additionally, because these tests were not developed to predict bleeding risk [59,60], it is unlikely that their use will contribute to the identification of safer anticoagulant compounds than the current ones. Therefore, the study of anticoagulant effects in orchids should be addressed through global coagulation tests. A thromboelastography analysis in whole blood would provide a better overview of the effects of the bioactive compounds on blood coagulation, evaluating simultaneously the activity of platelets, coagulation, and fibrinolytic factors [61]. Meanwhile, the thrombin generation assay would demonstrate whether the natural compounds’ in vitro anticoagulant activity reduces thrombin generation [62].

## 4. Future Directions

Either arterial or venous thrombosis affects almost all fields of current clinical medicine. Acute myocardial infarction, ischemic stroke, peripheral artery disease, deep vein thrombosis, and pulmonary embolism are the most recognized types of thrombosis. However, today, disseminated intravascular coagulation, splanchnic vein thrombosis, cerebral venous sinus thrombosis, and deep vein thrombosis of the upper extremities are some of the newer examples of thrombotic events gaining more and more clinical attention. Recent information shows that almost six out of ten human beings will suffer thrombosis during their life, a fact that may be translated into the highest economic burden for all health systems around the world [12,13]. Based on these facts, it is quite clear that any search to increase the prevention of thrombosis or to improve the current treatments is a priority for basic, epidemiological, and clinical research. Pharmacology, from all its fields, cannot be out of this integral research point of view, and this paper is a clear attempt at a search for new alternatives to obtain new antithrombotic agents.

It is important that research into natural compounds from orchids and other plants is focused on addressing the challenges of antithrombotic treatment. The balance between the safety and effectiveness of antithrombotic drugs may depend on the impact of these drugs on hemostasis and thrombosis, and inevitably, this involves developing drugs with specific mechanisms of action. In the field of anticoagulants, a new strategy to increase the safety of this treatment is to act on factor XI (FXI) [63]. The development of FXI inhibitors was based on mechanistic considerations that suggest a role of FXI in thrombus formation without significantly affecting hemostasis [64]. Interestingly, patients with congenital FXI deficiency have a lower risk of venous thromboembolism and ischemic stroke than non-deficient individuals and do not have an increased risk of spontaneous bleeding, even with severe deficiency [65]. This suggests that FXI inhibition could be a safety strategy for patients with thrombosis. According to this, the study of natural anticoagulants from orchids and other plants may focus on this new vision to treat thrombosis.

On the other hand, given that current antiplatelet drugs inhibit processes critical to hemostasis, it is logical to think that increasing the intensity of antiplatelet therapy will be associated with a higher bleeding risk. Over the past decade, it has been reported that the developing thrombus comprises two distinct regions, the hemostatic plug and the propagation thrombus [66]. The hemostatic plug is in the arterial lesion and is composed of fibrin and a core of activated platelets. Meanwhile, the propagation region is composed of platelets in a low-activation state and their recruitment appears to be minimally sensitive to commonly used antiplatelet agents [66,67]. This is important because knowledge of the factors that regulate thrombus propagation will generate new therapeutic approaches with benefits in thrombosis without apparent effects on hemostasis. Some of these factors have been identified [52]. Given this vision, it is understandable why for each natural compound with antiplatelet activity, its mechanism of action must be known.

## 5. Conclusions

This review supports scientific evidence that orchids are a natural source of compounds with biological properties that could be used to develop new antiplatelet, anticoagulant, and fibrinolytic drugs. Although the greatest evidence for this research field lies in platelet inhibition, anticoagulant and fibrinolytic effects are also quite desirable. Among several relevant findings, this review highlights that phenanthrenes are the most frequently isolated compounds and that *G. elata* has all three effects evaluated. However, the review revealed some gaps that must be addressed in the future, such as studying the mechanisms of action and the development of in vivo studies. The mechanisms of action are imperative because future approaches in this field must be directed to the specific inhibition of thrombus development but not hemostasis, in other words, to increase the efficacy (less thrombosis) while maintaining security (less hemorrhage).

## 6. Methods

We performed a selective narrative review of articles mainly written in English, but other languages were accepted. To this end, we searched for articles that included the combinations of different terms: orchids, antithrombotic activity, thrombosis, platelet aggregation, antiplatelet, anticoagulant, prothrombin time, activated thromboplastin time, thrombin time, thrombolytic activity, and fibrinolytic activity, among others. Searching for one article frequently led us to the next and so on. Searches without restriction on the publication date were conducted in PubMed, Scopus, Google Scholar, Web of Science, and directly on the Google page. We analyzed the available evidence that suggests antithrombotic activity of orchids, excluding those articles in which the antithrombotic effect was evaluated by mixing the extract of an orchid with that of another plant. Fifteen articles were finally included, all related to the antiplatelet, anticoagulant, and fibrinolytic activity of orchids.

## Figures and Tables

**Table 1:** Orchids with antithrombotic activity.

Orchid	Compound, Extract or Fraction	Chemical Class	Assay	Main Findings	Ref.
*Dendrobium loddigesii* Rolfe.	Moscatilin	Phenanthrene	In vitro; rabbit platelets	100 µg/mL: decreases PA with AA (0 ± 0% vs. 84.0 ± 6.7%), collagen (0 ± 0% vs. 86.4 ± 3.6%) and PAF (24.7 ± 2.9% vs. 86.3 ± 2.7%) vs. control (0.5% DMSO). IC_50_ = 61.8 µg/mL for AA-induced PA. IC_50_: 61.8 µg/mL for AA-induced PA.	[28]
	Moscatin			100 µg/mL: decreases PA with AA (0 ± 0% vs. 84.0 ± 6.7%), collagen (0 ± 0% vs. 86.4 ± 3.6%), thrombin (41.0 ± 9.5% vs. 89.3 ± 1.9%) and PAF (24.7 ± 2.9% vs. 86.3 ± 2.7%) vs. DMSO. IC_50_ = 37.2 µg/mL for AA-induced PA. IC_50_: 37.2 µg/mL for AA-induced PA.	
*Dendrobium densiflorum* Lindl.	Gigantol	Stilbenoid	In vitro; rat platelets	100 µM: decreases PA with ADP vs. control (29 ± 10% vs. 42 ± 5%). Inhibition = 31%.	[29]
	Moscatilin	Phenanthrene		Decreases PA with ADP vs. control. 50 µM: 30 ± 4% vs. 42 ± 5%; 100 µM: 27 ± 8% vs. 42 ± 5%. Inhibition = 29% (50 µM) and 36% (100 µM).	
	Homoeriodictyol	Flavone		Decreases PA with ADP vs. control. 50 µM: 35 ± 1% vs. 42 ± 5%; 100 µM: 21 ± 8% vs. 42 ± 5%. Inhibition = 17% (50 µM) and 50% (100 µM).	
	Scoparone	Coumarin		Decreases PA with ADP vs. control. 50 µM: 29 ± 19% vs. 42 ± 5%; 100 µM: 15 ± 11% vs. 42 ± 5%. Inhibition = 31% (50 µM) and 64% (100 µM).	
	Scopoletin			Decreases PA with ADP vs. control. 50 µM: 40 ± 8% vs. 42 ± 5%; 100 µM: 33 ± 5% vs. 42 ± 5%. Inhibition = 5% (50 µM) and 21% (100 µM).	
*Dendrobium longicornu* Lindl.	Longicornuol A (threo-1-{9-hydroxy-3-[2-(3-hydroxyphenyl)-ethyl]-1,8,10-trimethoxy- 6H-benzo[c]chromen-6-yl}-ethane-1,2-diol)	Bibenzyl	In vitro; rabbit platelets	1525.6 µM: inhibits PA with ADP in 73.59% vs. 100% of the positive control (1088.4 µM Ticlopidine). Ticlopidine (TCP).	[30]
	4-[2-(3-Hydroxyphenol)-1-methoxyethyl]-2,6-dimethoxyphenol	Bibenzyl		2348.7 µM: inhibits PA with ADP in 83.48% vs. 100% of TCP.	
	5-Hydroxy-7-methoxy-9,10-dihydrophenanthrene-1,4-dione	Phenanthrene		2789.1 µM: inhibits PA with ADP in 83.30% vs. 100% of TCP.	
	2,5,7-Trihydroxy-4-methoxy-9,10-dihydrophenanthrene	Phenanthrene		2767.4 µM: inhibits PA with ADP in 63.16% vs. 100% of TCP.	
	Erythro-1-(4-O-β-D-Glucopyranosyl-3-methoxyphenyl)-2-[4-(3-hydroxypropyl)-2,6-dimethoxyphenoxy]-1,3-propanediol	Lignin glycoside		1252.6 µM: inhibits PA with ADP in 33.16% vs. 100% of TCP.	
*Dendrobium trigonopus* Rchb.f.	Trigonopol A	Bibenzyl	In vitro; rabbit platelets	1.434 × 10^−3^ M: inhibits PA with AA in 67.55% vs. 100% of the positive control (1.088 × 10^−3^ M TCP).	[31]
*Dendrobium venustum* Teijsm. & Binn.	Lusianthridin	Phenanthrene	In vitro; human platelets	Inhibits the ADP-induced PA at 0.05 to 0.4 μM, with similar values for the positive control (35% approximately) from 0.2 μM. Positive control: acetylsalicylic acid (ASA). IC_50_: 0.22 ± 0.046 mM. Inhibits the AA-induced PA at the range concentrations of 0.025 to 0.1 μM, with similar values for ASA (10% approximately) from 0.025 M. IC_50_: 0.02 ± 0.001 mM. Inhibits the collagen-induced PA at 0.2 to 0.4 μM, with similar values for ASA (15% approximately) at 0.4 μM. IC_50_: 0.14 ± 0.018 mM. Binds the cyclooxygenase-1 (COX-1) enzyme with a pi-donor hydrogen bond with Tyr-355, two hydrophobic bonds with Val-116, and Ile-89 and a hydrophobic bond with Leu-93. Arg-120 and Glu-524 surrounded the binding site of lusianthridin. Binds the cyclooxygenase-2 (COX-2) enzyme with two hydrogen bonds with Arg-44 and Asn-39 and three hydrogen bonds with Pro-153, Leu-152, and Cys-47. Its binding site existed near the amino acid residues Glu-465 and Arg-469. Inhibition is concentration-dependent, as well as COX-1 and COX-2 activities. The IC_50_ of lusianthridin for COX-1 and COX-2 are 10.81 ± 1.12 μM and 0.17 ± 1.62 μM, respectively. At 0.4 mM, it prevents the decrease of ADP-induced cAMP level (12.21 ± 1.42 pmol/10^8^ platelets) in a similar way as 3-isobutyl-1-methylxanthine (11.66 ± 1.98 pmol/10^8^ platelets). Positive control: 3-isobutyl-1-methylxanthine.	[32]
*Cleisomeria lanatum* (Lindl.) Lindl. ex. G.Don	Methanolic extracts of leaves, roots, and stems.	Alkaloids, flavonoids, saponins, tannins, phenols, terpenoids, steroids, glycosides, cardiac glycosides, quinine, coumarin, proteins, and resins	In vitro; human platelets	250 µg/mL of stem extract: showed a percentage of clot lysis activity of 47.2%. IC_50_: 163.8 µg/mL. 250 µg/mL of leaf extract: showed a percentage of clot lysis activity of 43.4%. IC_50_: 173.7 µg/mL. 250 µg/mL of root extract: showed a percentage of clot lysis activity of 32.5%, and IC_50_: 190.9 µg/mL.	[33]
*Ephemerantha lonchophylla* (Hook. f.) P.F. Hunt & Summerh.	Denbiobin	Phenanthrene	In vitro; rabbit platelets	100 µg/mL: decreases PA with thrombin (87.6 ± 1.2% vs. 92.6 ± 1.4%), AA (71.6 ± 2.2% vs. 85.9 ± 0.5%) and collagen (80.9 ± 2.6% vs. 88.1 ± 0.9%) vs. control (0.5% DMSO).	[34]
	3,7-dihidroxy-2,4-dimethoxyphenanthrene			5 µg/mL: decreases PA with AA (57.7 ± 9.8%) vs. DMSO (85.9 ± 0.5%). 10 µg/mL: decreases PA with AA (12.1 ± 6.7%) vs. DMSO (85.9 ± 0.5%). 20 µg/mL: decreases PA with AA (0.0 ± 0.0%) vs. DMSO (85.9 ± 0.5%). 50 µg/mL: decreases PA with AA (0.0 ± 0% vs. 85.9 ± 0.5%) and PAF (75.4 ± 3.7% vs. 88.1 ± 0.7%) vs. DMSO. 100 µg/mL: decreases PA with AA (0.0 ± 0% vs. 85.9 ± 0.5%; IC_50_: 24 µM), collagen (11.8 ± 1.7% vs. 88.1 ± 0.9%) and PAF (7.0 ± 2.8% vs. 88.1 ± 0.7%) vs. DMSO. Note: this compound suppressed AA-induced PA at a lower concentration (20 µg/mL) than the positive control (50 µg/mL Acetylsalicylic acid).	
	Erianthridin			2 µg/mL: decreases PA with AA (54.1 ± 14.0%) vs. DMSO (85.9 ± 0.5%). 5 µg/mL: decreases PA with AA (10.9 ± 4.3) vs. DMSO (85.9 ± 0.5%). 10 µg/mL: decreases PA with AA (0.0 ± 0%) vs. DMSO (85.9 ± 0.5%). 100 µg/mL: decreases PA with AA (0.0 ± 0% vs. 85.9 ± 0.5%; IC_50_: 9 µM), thrombin (86.3 ± 0.4% vs. 92.6 ± 1.4%), collagen (16.3 ± 1.8% vs. 88.1 ± 0.9%), and PAF (34.2 ± 5.1% vs. 88.1 ± 0.7%) vs. DMSO. Note: this compound suppressed AA-induced PA at a lower concentration (10 µg/mL) than the positive control (50 µg/mL ASA).	
	3-methylgigantol	Stilbenoid		5 µg/mL: decreases PA with AA (69.1 ± 7.6%) vs. DMSO (85.9 ± 0.5%). 10 µg/mL: decreases PA with AA (35.1 ± 14.8%) vs. DMSO (85.9 ± 0.5%). 20 µg/mL: decreases PA with AA (10.7 ± 5.4% vs. 85.9 ± 0.5%) and PAF (80.6 ± 3.6 vs. 88.1 ± 0.7%) vs. DMSO. 50 µg/mL: decreases PA with AA (0.0 ± 0% vs. 85.9 ± 0.5%) and PAF (62.1 ± 4.2% vs. 88.1 ± 0.7%) vs. DMSO. 100 µg/mL: decreases PA with AA (0.0 ± 0% vs. 85.9 ± 0.5%; IC_50_: 30 µM), collagen (4.2 ± 2.0% vs. 88.1 ± 0.9%) and PAF (1.4 ± 1.2% vs. 88.1 ± 0.7%) vs. DMSO.	
*Calanthe arisanensis* Hayata.	2,7-dihydroxy-3,5-dimethoxy-9,10-dihydro-phenanthrene	Phenanthrene	In vitro; human platelets	Inhibits the collagen-induced PA more potently than positive control (IC_50_: 0.2 µg/mL vs. 13.8 µg/mL). Positive control: ASA.	[35]
	2,7-dihydroxy-4-methoxy-9,10-dihydro-phenanthrene			Inhibits the collagen-induced PA more potently than ASA (IC_50_: 1.3 µg/mL vs. 13.8 µg/mL).	
	Compound 9			Inhibits the collagen-induced PA more potently than ASA (IC_50_: 10.7 µg/mL vs. 13.8 µg/mL).	
	Compound 11			Inhibits the collagen-induced PA more potently than ASA (IC_50_: 3.4 µg/mL vs. 13.8 µg/mL).	
	Compound 12			Inhibits the collagen-induced PA more potently than ASA (IC_50_: 8.9 µg/mL vs. 13.8 µg/mL).	
	Compound 13			Inhibits the collagen-induced PA more potently than ASA (IC_50_: 4.0 µg/mL vs. 13.8 µg/mL).	
	Compound 14			Inhibits the collagen-induced PA more potently than ASA (IC_50_: 1.8 µg/mL vs. 13.8 µg/mL).	
	Compound 15			Inhibits the collagen-induced PA more potently than ASA (IC_50_: 2.6 µg/mL vs. 13.8 µg/mL).	
	Compound 16			Inhibits the collagen-induced PA more potently than ASA (IC_50_: 2.4 µg/mL vs. 13.8 µg/mL).	
*Cephalanceropsis gracilis* (Lindl.) S.Y. Hu.	Cephalandole A 2	Alcaloide indol	In vitro; rabbit platelets	Inhibits the AA-induced PA more potently than ASA (IC_50_: 19.34 ±0.27 µg/mL vs. 22.17 ± 0.51 µg/mL). Positive control: ASA.	[36]
*Gastrodia elata* Blume.	Methanol (II), ethyl acetate (III), and butanol (IV) extract	The compounds were not identified	Mouse and rat thrombosis models	Fractions II, III, and IV, at 2.5 mg/mL, suppressed the ADP- and collagen-induced PA. Oral administration of 100 mg/kg of fractions II, III and IV in mice induced recovery from thrombotic paralysis by 40, 44, and 32%, respectively. Oral administration of 500 mg/kg of fractions II, III, and IV in rats reduced the effects of intravenous administration of collagen (60 µg/10 mL saline/kg) on platelet count. For fractions II, III, and IV, the mean percentage reduction in platelet count was 5.4%, 13.7%, and 10%, respectively. For the control (collagen), the reduction was 15%.	[37]
	Fraction G2	The compounds were not identified	In vitro; human platelets	0.3 mg/mL: decreased the ADP-induced PA vs. control (35.2 ± 7.7% vs. 49.7 ± 7.4%). 1.2 and 2.4 mg/mL: decreased PAF-induced PA vs. control (38.6 ± 14.3% vs. 66.9 ± 10.9%; 6.2 ± 7.5% vs. 66.9 ± 10.9%, respectively). 2.4 mg/mL: decreased the ADP-induced PA vs. control (11.2 ± 6.5% vs. 49.7 ± 7.4%). The AA-induced PA was lower than the control (72.0 ± 9.5% vs. 20.6 ± 19.8%).	[38]
			In vivo; rats	150, 200, 250 mg/kg: decreased ADP-induced PA vs. control (41.1 ± 15.8%, 46.7 ± 10.8%, 47.6 ± 15.0%, respectively vs. 61.5 ± 8.2%).	
			In vivo model of acute pulmonary embolism in mice	5 and 10 mg/kg: decreased the percentage of dead mice due to thrombosis induced by ADP (300 mg/kg) vs. control (26.7% and 15.4% vs. 41.7%, respectively).	
	4,4′-dihydroxybenzilsulfone	Phenol (sulfone)	In vitro; rat platelets	Inhibits the U46619-induced PA more potently than positive control (IC_50_: 83 µM vs. 340 µM). Positive control: ASA.	[39]
	4-[4′-(4″-hydroxybenzyloxy) benzyloxy]benzyl methyl ether	Phenol (Phenyl ethers)		Inhibits the collagen-induced PA more potently than ASA (IC_50_: 191 µM vs. 420 µM).	
	4,4′-dihydroxy-diphenyl methane	Phenol (diphenyl methano)		Inhibits the AA-induced PA more potently than ASA (IC_50_: 30 µM vs. 66 µM).	
	4,4-dihydroxy-dibenzylether	Phenol (dibenzylether)		Inhibits the PA induced by collagen (5 µM vs. 420 µM), EPI (3 µM vs. 53 µM), and U46619 (33 µM vs. 340 µM) more potently than ASA.	
	Polysaccharide 2-1:	Polysaccharide	In vivo; mice	30 mg/kg: prolongs BT (12.05 ± 1.5 min vs. 9.86 ± 0.82 min) and RT (7.62 ± 0.31 A_540_ vs. 6.10 ± 0.03 A_540_) vs. control (saline solution). 60 mg/kg: prolongs BT (13.76 ± 1.91 min vs. 9.86 ± 0.82 min), CT (6.25 ± 0.43 min vs. 4.15 ± 0.22 min) and BC (0.40 ± 0.07 vs. 0.14 ± 0.04 A_540_) vs. control. 120 mg/kg: prolongs BT (16.51 ± 2.89 min vs. 9.86 ± 0.82 min), CT (8.19 ± 0.25 min vs. 4.15 ± 0.22 min) and BC (0.31 ± 0.06 vs. 0.14 ± 0.04 A_540_) vs. control. Inhibits ADP-induced PA, mainly at 120 mg/kg. Note: despite the effects of PGE2-1 on CT, BT, BC, and RT, none of these were greater than those of heparin (positive control).	[40]
			Ex vivo; human plasma	10 mg/mL: prolongs TT (11.33 ± 0.80 s) vs. control (7.85 ± 0.64 s). 20 mg/mL: prolongs TT (11.48 ± 1.16 s vs. 7.85 ± 0.64 s) and APTT (41.40 ± 2.10 s vs. 33.17 ± 3.19 s) vs. control. 40 mg/mL: prolongs APTT (45.07 ± 3.11 s) vs. control (33.17 ± 3.19 s). Note: PGE2-1 had a lower anticoagulant effect than heparin.	
			In vivo (mice); thrombosis induced by ADP	60 mg/kg: shortens recovery time (16.62 ± 1.78 min) vs. heparin (18.28 ± 1.65 min). 120 mg/kg: shortens recovery time (11.27 ± 1.02 min) vs. heparin (18.28 ± 1.65 min).	
			In vitro (blood); rats treated with PGE2-1	120 mg/kg: decreases the length of the thrombus (1.73 ± 0.41 cm vs. 4.68 ± 0.79 cm), wet weight of the thrombus (130.68 ± 11.06 mg vs. 230.60 ± 40.86 mg), and dry weight of the thrombus (22.0 ± 3.61 mg vs. 56.83 ± 8.71 mg) vs. control.	
			In vivo (arteriovenous shunt thrombosis); rats treated with PGE2-1.	30 mg/kg: decreases the wet weight of the thrombus (59.75 ± 2.65 mg) vs. untreated rats (88.50 ± 2.82 mg). Inhibition percentage: 32.5%. 60 mg/kg: decreases the wet weight of the thrombus (44.78 ± 3.76 mg) vs. untreated rats (88.50 ± 2.82 mg). Inhibition percentage: 49.0% 120 mg/kg: decreases the wet weight of the thrombus (34.10 ± 4.56 mg) vs. untreated rats (88.50 ± 2.82 mg). Inhibition percentage: 61.5%.	
	4-Hydroxybenzyl alcohol	Phenol (benzyl alcohol)	In vitro; plasmin activity	Increased plasmin activity by more than 100% vs. positive control (plasmin 50 nM)	[41]
	4-Hydroxybenzaldehyde	Phenol (benzaldehyde)		Increased plasmin activity by approximately 25% vs. plasmin.	
	4-Ethoxymethyl phenol	Phenol (ethoxymethyl)		Increased plasmin activity by approximately 50% vs. plasmin.	
*Laelia furfuracea* Lindl.	Foliar hydroethanolic extract and ethyl acetate fraction	Carboxylic acid, phenolic acid, flavonoids, flavone, and terpene	Ex vivo; human plasma from thrombotic patients	25 mg/mL: prolongs PT (49.1 ± 0.8 s vs. 13.7 ± 0.3 s) and TT (30.1 ± 0.9 s vs. 16.6 ± 0.5 s) vs. basal condition. 15 mg/mL: prolongs APTT vs. basal condition (61.8 ± 3.4 s vs. 32.0 ± 1.2 s). Ethyl acetate fraction prolongs by more than 300 s the TT, PT, APTT at final concentrations of 30, 28, and 15 mg/mL, respectively.	[42]

AA: arachidonic acid; ADP: adenosine diphosphate; APTT: activated partial thromboplastin time; ASA: acetylsalicylic acid; BC: bleeding capacity; BT: bleeding time; CT: coagulation time; RT: recalcification time; COX-1: cyclooxigenase-1; COX-2: cyclooxigenase-2; DMSO: dimethyl sulfoxide; EPI: epinephrine; IC_50_: inhibitory concentration 50; PA: platelet aggregation; PAF: platelet activator factor; PGE2-1: polysaccharide of *Gastrodia elata* 2-1; PT: prothrombin time; TCP: ticlopidine, TT: thrombin time.

## Data Availability

Not applicable.
